# Intravenous morphine versus intravenous paracetamol after cardiac surgery in neonates and infants: a study protocol for a randomized controlled trial

**DOI:** 10.1186/s13063-018-2705-5

**Published:** 2018-06-13

**Authors:** Gerdien A. Zeilmaker-Roest, Joost van Rosmalen, Monique van Dijk, Erik Koomen, Nicolaas J. G. Jansen, Martin C. J. Kneyber, Sofie Maebe, Greet van den Berghe, Dirk Vlasselaers, Ad J. J. C. Bogers, Dick Tibboel, Enno D. Wildschut

**Affiliations:** 1 Department of Pediatric Intensive Care, Erasmus MC-Sophia Children’s Hospital, Rotterdam, The Netherlands; 2 Department of Cardiothoracic Surgery, Erasmus MC, Rotterdam, The Netherlands; 3 Department of Biostatistics, Erasmus MC, Rotterdam, The Netherlands; 4 Department of Pediatric Intensive Care, Wilhelmina Children’s Hospital, University Medical Center Utrecht, Utrecht, The Netherlands; 5 Department of Pediatrics, division of Pediatric Critical Care Medicine, Beatrix Children’s Hospital, University Medical Center Groningen, Groningen, The Netherlands; 6 Department of Intensive Care Medicine, UZ Leuven, Leuven, Belgium

**Keywords:** Pain, Sedation, Opioids, Children, Intensive care, Cardiac surgery, PK, PD

## Abstract

**Background:**

Morphine is worldwide the analgesic of first choice after cardiac surgery in children. Morphine has unwanted hemodynamic and respiratory side effects. Therefore, post–cardiac surgery patients may potentially benefit from a non-opioid drug for pain relief. A previous study has shown that intravenous (IV) paracetamol is effective and opioid-sparing in children after major non-cardiac surgery. The aim of the study is to test the hypothesis that intermittent IV paracetamol administration in children after cardiac surgery will result in a reduction of at least 30% of the cumulative morphine requirement.

**Methods:**

This is a prospective, multi-center, randomized controlled trial at four level-3 pediatric intensive care units (ICUs) in the Netherlands and Belgium. Children who are 0–36 months old will be randomly assigned to receive either intermittent IV paracetamol or continuous IV morphine up to 48 h post-operatively. Morphine will be available as rescue medication for both groups. Validated pain and sedation assessment tools will be used to monitor patients. The sample size (*n* = 208, 104 per arm) was calculated in order to detect a 30% reduction in morphine dose; two-sided significance level was 5% and power was 95%.

**Discussion:**

This study will focus on the reduction, or replacement, of morphine by IV paracetamol in children (0–36 months old) after cardiac surgery. The results of this study will form the basis of a new pain management algorithm and will be implemented at the participating ICUs, resulting in an evidence-based guideline on post-operative pain after cardiac surgery in infants who are 0–36 months old.

**Trial registration:**

Dutch Trial Registry (www.trialregister.nl): NTR5448 on September 1, 2015. Institutional review board approval (MEC2015–646), current protocol version: July 3, 2017

**Electronic supplementary material:**

The online version of this article (10.1186/s13063-018-2705-5) contains supplementary material, which is available to authorized users.

## Background

Congenital heart disease accounts for almost one third of all congenital anomalies, and the reported total prevalence in Europe is 8.0 in 1000 births [1, 2]. Surgical intervention is necessary in 55% within the first year of life and in 67% during the first three years of life [3].

The importance of adequate post-surgical pain relief in neonates and infants became apparent after findings that untreated pain results in increased stress hormone levels and prolonged behavioral consequences [4]. These findings have resulted in an increased use of morphine as the worldwide standard for pain relief after major surgery in neonates and children [5–9]. However, morphine can cause unwanted hemodynamic and respiratory reactions and therefore patients could potentially benefit from a non-opioid analgesic.

In a recent randomized controlled trial, intravenous (IV) paracetamol was compared with morphine as a primary analgesic drug in non-cardiac post-operative children up to 1 year. IV paracetamol was equally effective in pain relief, and no difference in rescue analgesics was shown between groups [10]. The IV paracetamol group had a lower cumulative morphine dose the first 48 h after surgery and less adverse drug reactions. Whether these results also apply to neonates and children after cardiac surgery is unclear. Pharmacokinetic (PK) parameters are assumed to be different in patients during and after cardiac surgery compared with non-cardiac surgery, and changes in pharmacodynamics (PD) and pharmacogenetics concerning pain perception need to be taken into account.

### Cardiothoracic surgery

Based on the general anesthesia guidelines, opioids are considered standard of care in children to prevent and treat post-operative pain after cardiac surgery even though clear PK data are lacking [11]. There are reasons to assume that children after cardiac surgery have different PK or PD (or both) compared with adults or children after non-cardiac surgery. Valkenburg et al. describe a lower clearance of morphine and a higher volume of distribution in 38 children after cardiac surgery compared with non-cardiac surgery, possibly necessitating a different dosing regimen [12].

The use of cardiopulmonary bypass (CPB) is the main reason to expect PK changes. CPB has a profound effect on the PK parameters because of hemodynamic changes, hemodilution, hypothermia, and systemic inflammatory reactions (systemic inflammatory response syndrome, or SIRS). These effects change constantly throughout CPB and some continue to exert an influence after the patient has been successfully weaned from CPB [13].

Hemodynamic changes affect organ perfusion and ultimately organ function. Hemodynamics may also be altered by the need for inotropic support during and after surgery. Children with a normal cardiovascular system undergoing surgery seem to clear morphine more efficiently than infants undergoing cardiovascular surgery [14]. On initiation of CPB, prime fluid causes dilution of the patient’s blood. This causes a shift in the bound and unbound fraction of the drug and a redistribution from peripheral to central compartments. Decreased renal and hepatic perfusion due to hypothermia, hypotension, decreased flow rate, and hemodilution may result in decreased elimination of drugs [13].

Surgical correction of more complex congenital cardiac defects has an additional influence on systemic circulation influencing the PK of drugs itself because of longer CPB run times with higher risk for severe SIRS and hemodynamic instability [13, 15, 16]. However, there is currently no literature suggesting a difference in sedative or analgesic requirements based on type of congenital defect or surgical procedure.

### Morphine

The elimination of morphine is mainly through glucuronidation by urine diphosphate glucuronosyltransferase (UGT) 2B7. Morphine clearance directly reflects the formation of its two major metabolites that are pharmacologically active: morphine-3-glucuronide (M3G) and morphine-6-glucuronide (M6G). Both metabolites are cleared through renal elimination, and a reduced renal function can lead to accumulation. Evidence suggests that clearance of morphine is significantly slower in children who need inotropic support after cardiac surgery [17].

The unwanted hemodynamic and respiratory effects of morphine are a particular problem in these hemodynamically unstable children and may lead to delayed recovery and prolonged pediatric intensive care unit (PICU) stay [18, 19]. Another adverse effect of morphine is intestinal obstruction, which occurs mainly in younger children, whereas nausea, vomiting, and itching occur mainly in older children [7].

### Paracetamol

The analgesic effect of paracetamol is not yet fully understood but is likely due to interaction with the serotonergic system. Glucuronidation is the major pathway of paracetamol metabolism (50–60%) and there is a contribution of sulfation (25–44%) and oxidation (2–10%). Glucuronidation and sulfation result in inactive and non-toxic end products. The hepatic oxidation pathway forms NAPQI (N-acetyl-p-benzo-quinone imine) [20]. NAPQI is toxic and, in the case of overdose, causes mitochondrial dysfunction and centrilobular necrosis in the liver [21]. Several studies show that, when it is used in therapeutic doses in children without liver dysfunction, the safety profile is excellent [20].

Enteral or rectal dosing is sufficient for mild to moderate pain. However, after major surgery, rectal administration of paracetamol was shown to be insufficient to reach a therapeutic level for pain relief and does not reduce the morphine consumption [22–24]. Potentially, IV paracetamol performs better in the case of severe or acute pain. IV paracetamol rapidly penetrates an intact blood-brain barrier in children, which contributes to the fast onset of the analgesic effect [25, 26, 27].

### Pharmacogenetics

A large number of candidate gene studies have illustrated associations between genetic variants with opioid response [28, 29] and paracetamol efficacy [30]. The genetic impact can arise from polymorphisms in genes that alter drug levels (PK) such as metabolizing enzymes and transporters. PK genes relevant for morphine are *UGT2B7*, *ABCC3*, and *OCT1* [31–33].

### Hypothesis

Our hypothesis is that intermittent IV paracetamol is effective as the primary analgesic drug in post-cardiac surgery patients up to 3 years of age and that the use of IV paracetamol will reduce overall morphine requirements.

This hypothesis is currently being tested at three level-4 PICUs in the Netherlands and Belgium (Erasmus MC-Sophia Rotterdam, Wilhelmina Children’s Hospital University Medical Center (UMC) Utrecht, Beatrix Children’s Hospital UMC Groningen, University Hospital (UZ) Leuven).

## Methods

### Trial design

This study is a multi-center, prospective, randomized, double-blind study with a non-inferiority design.

### Study setting

The study will be conducted at four level-3 PICUs in the Netherlands and Belgium (Erasmus MC-Sophia Rotterdam, Wilhelmina Children’s Hospital UMC Utrecht, Beatrix Children’s Hospital UMC Groningen and UZ Leuven).

### Interventions

All patients will receive a loading dose of morphine 100 μg/kg at the end of surgery. After the loading dose, patients are randomly assigned to receive either a morphine continuous infusion or intermittent IV paracetamol. A double dummy (intermittent or continuous infusion of NaCl 0.9%) will be used in each group to ensure blinding. Patients in the intervention group receive intermittent IV paracetamol.

### Justification of dosing

Howard et al. prospectively evaluated effectiveness, morphine requirements, and safety of IV morphine in over 10,000 pediatric patients, including almost 1000 patients after cardiac surgery in a tertiary care hospital [34]. Patients after cardiac surgery had the highest morphine requirements—an average dose of 23 μg/kg per h (5–50 μg/kg per h)—but PK data were not collected and it is unclear whether morphine dosing was based on validated pain scores. Lynn et al. described morphine serum levels of 15–20 ng/mL in infants after cardiac surgery with continuous morphine infusions of 10–20 μg/kg per h without describing analgesic efficacy [9]. These morphine serum levels are typically associated with adequate pain relief [17]. In 2009, Knibbe et al. proposed a novel morphine dosing regimen for neonates and children on the basis of PK studies, resulting in a significant dose reduction of morphine in neonates of less than 10 days post-natal age [35]. This model was further validated with new datasets [36] and ultimately resulted in a prospective randomized controlled trial in which the proposed dosing regimen for morphine dosing was evaluated [37]. This morphine dosing regimen was used in the previous trial comparing continuous IV morphine with intermittent IV paracetamol after major non-cardiac surgery.

Several studies have determined paracetamol metabolism in children [38, 20]. PK of IV paracetamol in children until the age of 16 years have been described; however, no children undergoing cardiac surgery were included in this study [39, 40].

IV paracetamol will be dosed in accordance with the Dutch pediatric formulary with a loading dose of 20 mg/kg and maintenance dose of 10 mg/kg (<1 month of age) or 15 mg/kg (>1 month) [41].

Worldwide, IV paracetamol is given in three or four dosages daily and not as continuous infusion. Even though continuous administration of IV paracetamol is possible, limited evidence in healthy adults shows that the analgesic effect of IV paracetamol is better with intermittent administration [42]. Therefore, different delivery schedules for morphine (continuous) and paracetamol (intermittent) are selected.

### Control group

Patients in the control group will receive a continuous morphine infusion after the loading dose of morphine 100 μg/kg. Using our PK data on morphine in non-cardiac and cardiac pediatric patients [35, 37, 40], we developed a new morphine dosing regimen for neonates and children who are 0 to 36 months old. The dosing algorithm is presented as Table 1, in the Appendix.

### Use of co-intervention

Standard post-operative care is given to all patients with analgesic rescue medication. Short-acting analgesics and sedatives are available during interventions, such as chest drain removal. All co-medications used during the first 48 h after surgery are registered in the database.

### Rescue analgesic medication

Rescue morphine will be administered with a maximum of three times per hour whenever the Numeric Rating Scale (NRS) score is at least 4. Standard additional dose of morphine is 10 μg/kg for patients who are less than 10 days post-natal age and 15 μg/kg for patients who are at least 10 days.

Ten minutes after each extra dose of morphine, pain is re-assessed. If there is no improvement in the scores (i.e., NRS score ≥4) after three additional (rescue) doses, a morphine loading dose of 100 μg/kg is given and a continuous morphine infusion is started at 10 μg/kg per h in a separate pump (to ensure blinding). Whenever pain is not responding to the extra morphine boluses and the additional continuous morphine infusion in a maximum dose of 30 μg/kg per h, fentanyl is started at 1–2 μg/kg loading dose and 1–3 μg/kg per h continuous infusion. At the start of fentanyl, morphine will be discontinued. In case of discomfort, midazolam is started. Discomfort is determined as COMFORT-Behavior scale (COMFORT-B) score of more than 22 or COMFORT-B score between 11 and 22 but with the Nurse Interpretation of Sedation Score (NISS) suggesting undersedation. Standardized sedation will be part of the treatment protocol. Sedation protocols regarding the primary sedatives are already comparable between the participating ICUs. This treatment algorithm is similar to the one used in the recent study comparing morphine and paracetamol in non-cardiac patients [10].

In both groups, continuous morphine infusion (if started) will be decreased in the second 24 h depending on the NRS and COMFORT-B scores.

If discharge from the ICU occurs within 48 h after surgery, the study medication will be continued on the ward. The arterial catheter will be removed at discharge from the ICU. PK sampling on the ward will only be carried out simultaneously with routine blood examination. At 48 h after surgery, the study medication will be changed to open-label paracetamol and rescue morphine if needed.

### PD outcome measurements

PD outcomes will be measured by using validated instruments. Both pain and under- and over-sedation need to be assessed. Signs of pain and distress may overlap, making accurate assessment difficult. Therefore, the use of concomitant sedative drugs will be standardized in the participating ICUs. The COMFORT-B is mainly a distress assessment and, to a lesser extent, a pain instrument that asks observers to consider the intensity of six behavioral manifestations: alertness, calmness, respiratory response (for mechanically ventilated children) or crying (for spontaneously breathing children), body movements, facial tension, and muscle tone. For each of these items, five descriptions, rated from 1 to 5, reflecting increasing intensity of the behavior in question, are provided. Summating the ratings of the six behavioral manifestations leads to a total score ranging from 6 to 30. The COMFORT-B has been extensively validated in post-operative infants with or without Down syndrome and in infants after major cardiac surgery [43–46].

The NRS for pain is a scale from 0 (no pain) to 10 (worst possible pain) and is used in conjunction with the COMFORT-B to represent the rater’s expert opinion [47]. The NRS focusses on pain, whereas the COMFORT-B assesses mainly discomfort. Any additional rescue morphine is given when the NRS score is 4 or higher. This dose escalation schedule is consistent with additional analgesic treatment in the normal clinical setting.

Using the NRS, parents will be able to participate in rating the pain in their children. This will be used alongside the nurses’ NRS and COMFORT-B. Parents will receive a very short instruction explaining the different factors that should be taken into account when assigning the NRS.

Parents’ participation will be on a voluntary basis. If parents indicate that active participation is not wanted anymore, they will be able to stop at any time. Variation in nurse and parent evaluation of pain or discomfort will be analyzed as a secondary endpoint.

The NISS has been validated for this age group and represents the caregiving nurse’s expert opinion and is scored as 1 = undersedation, 2 = adequate sedation, or 3 = oversedation [47].

Follow-up will consist of the “Parents’ Postoperative Pain Measurement–Short Form” (PPPM-SF) to complete after discharge from the hospital. This tool includes 10 items asking parents about signs of pain and distress at home in children after surgical intervention [48]. Parents will be called 2 d after discharge from the hospital to inform after the child’s health and to take the questionnaire.

Patients after major cardiac surgery are also at risk for opioid withdrawal syndrome and pediatric delirium. Therefore, these two conditions were assessed as well [33, 49].

The Sophia Observation withdrawal Symptoms (SOS) scale has been validated to detect withdrawal syndrome in critically ill children. It contains 15 items that are scored as either not present (0) or present (1). A score of 4 or higher suggests withdrawal syndrome [50, 51].

Pediatric delirium in our study will be assessed by using the SOS-PD scale and Cornell Assessment of Pediatric Delirium (CAPD) [52]. The SOS-PD scale consists of 17 items and the maximum total sum score is 17 points. A total score of at least 4 is used as a cutoff for delirium or when the item “hallucination” is scored positive. The CAPD consist of eight items, scoring the interaction of the nurse with the patient. The maximum total sum score is 32 points, and the cutoff for delirium is 9 points or greater. In accordance with standard local protocol, a pediatric psychiatrist will be consulted in case of delirium. Treatment of delirium will be carried out in accordance with local protocol [53].

Severity of illness will be estimated with the validated Pediatric Risk of Mortality III score [54] together with the more specific Risk-Adjusted Classification for Congenital Heart Surgery (RACHS-1) score [55]. All Dutch hospitals use the Aristotle score to classify the congenital cardiac surgery patients. This Aristotle score will also be used to estimate the severity of surgical procedure and to compare the two groups with respect to the surgical intervention [56, 57]. The Pediatric Logistic Organ Dysfunction 2 (PELOD-2) score will be used to assess the severity of cases of multiple organ dysfunction syndrome in the PICU on day 0 (day of surgery), day 1, and day 2 [58].

### PK analysis and blood sampling

Blood samples will be drawn for PKPD analysis by using an indwelling arterial catheter. Blood samples will be taken after the morphine bolus dose, directly after the start of trial medication, 30–60 min after the start of trial medication, 3–4 h after the start of trial medication, and at three standard moments during the day. The timing of the standard sampling moments is dependent on local clinical practice. Because of small timing differences, this will create a diverse sampling scheme, which is very applicable for PKPD analysis. Also, samples will be taken before and after changes in the dose of analgesic medication. For ethical reasons, not more than 5% of the total blood volume will be drawn from the patient. Thus, PK samples will be obtained by sparse sampling with a minimal burden to the individual patients. Using population PK analysis, the data points from the individual subjects will be combined to form solid PK data on morphine and paracetamol and their metabolites since these metabolites are biologically active. The population PK analysis will be carried out by using non-linear mixed-effect modeling (NONMEM).

DNA analysis will be performed to evaluate interindividual variability drug responses. Genetic variability of morphine and paracetamol will be the main focus.

### Eligibility criteria and parental consent

Patients eligible to participate in the study will be infants and children (0–36 months) who are admitted to the ICU after cardiac surgery with the use of the CPB. Information regarding the trial will be given to parents, or authorized surrogates, of potential trial participants at the out-patient clinic or on the ward. Information will be given by a researcher, either the doctor or research nurse. Written informed consent is obtained from all trial participants before surgery.

#### Inclusion criteria

- Informed consent
- Neonates/infants who are 0–36 months old
- Cardiac surgery with the use of CPB.

#### Exclusion criteria

- No informed consent
- Known allergy to or intolerance of paracetamol or morphine
- Administration of opioids in the 24 h prior to surgery
- Hepatic dysfunction defined as three times the reference value of alanine aminotransferase/aspartate aminotransferase (ALAT/ASAT)
- Renal insufficiency defined as Pediatric RIFLE (Risk, Injury, Failure, Loss, End Stage Renal Disease) category; injury is defined as estimated creatinine clearance reduced by 50% and urine output of less than 0.5 mL/kg per h for 16 h.

If patients develop renal or hepatic insufficiency after randomization, they will be withdrawn from the trial.

### Primary objectives

The primary objective is to test the hypothesis that analgesia with intermittent IV paracetamol will lead to a morphine-sparing effect of at least 30% as compared with model-based continuous IV morphine infusion during the first 48 h after cardiac surgery in infants who are 0–36 months old.

The primary outcome measure is weight-adjusted cumulative morphine consumption (in micrograms per kilogram) in the first 48 h post-operatively.

The reduction of morphine has been chosen as the primary outcome since this is directly related with the decrease of morphine-related adverse drug reactions. The previous trial comparing IV paracetamol with morphine clearly showed a reduction of morphine-related adverse drug reactions in the IV paracetamol group. Among these drug reactions are gastrointestinal symptoms, which are also described as two out of six endpoints in the recent Standardised Endpoints in Perioperative Medicine (StEP) recommendations for patient comfort [59].

### Secondary objectives

1. Incidence of adverse drug reactions
  - Hemodynamic: hypotension or bradycardia, with the need for intervention by means of medication or a fluid bolus
  - Decreased gastrointestinal motility or intestinal obstruction not directly related to the underlying diagnosis and not previously existing, with the need for intervention
  - Vomiting
  - Number of re-intubations
  - Pediatric delirium as measured by the SOS-PD scale or CAPD score
2. Non-inferiority analysis of comparing patients with one or more NRS scores of at least 4 between groups
3. DNA analysis will be performed to evaluate the effect of gene polymorphisms on the PK of analgesic medication
4. Concomitant use of sedatives
5. The number of hours on ventilation
6. The length of PICU stay
7. Role of alarmins in the systemic inflammatory response (only at Wilhelmina Children’s Hospital, UMCUtrecht).
8. To develop a population PKPD-based post-operative pain management algorithm based on the results of this trial.

### Sample size calculation

We estimate that the required morphine dose in the paracetamol group can be reduced by at least 30% compared with the morphine group in the first 48 h after surgery. This is in line with the outcome of a previous study we conducted in a 0- to 1-year-old patient group after major non-cardiac surgery with similar study medication [10].

The power analysis is based on a comparison of the primary outcome between groups using a Mann-Whitney test. A simulation study was carried out for this power analysis using data on the cumulative morphine dose from a previous study with comparable morphine dosing [10]. Based on this dataset, the median cumulative morphine dose was 357 μg/kg (interquartile range of 220–605) in the control group, and we assumed that this morphine dose would be reduced by 30% in the intervention group. The simulation study showed that, with a two-sided significance level of 5%, 86 patients per group would be required to obtain a power of 95%. To account for the effects of stratification by center and missing data, we will include 104 patients per study arm (208 in total). We expect this sample size to be sufficient to assess secondary outcomes.

As described above, the primary endpoint of the study is the total amount of morphine administered in the first 48 h after surgery. Any additional morphine given will be based on NRS scores of 4 or higher. Therefore, in a secondary, non-inferiority analysis, we will also compare the percentage of patients with one or more NRS scores of 4 or higher between the two groups, using a non-inferiority margin of 20%, based on the previous trial and clinical experience. NRS scores are assessed often per patient and should give a clear indication of the number of painful moments per treatment group. Since NRS scores are recorded often per patient, we set the non-inferiority margin on 20%, assuming that this would reflect a clinically relevant difference. Non-inferiority will be assessed by using a one-sided 97.5% confidence interval for the difference in the percentage of patients with at least one NRS score of 4 or higher between the paracetamol group and the morphine group, and non-inferiority will be proven if the upper limit of this confidence interval is lower than 20%. The confidence interval will be calculated by using the method of Klingenberg [60] with adjustment for center. With data from our previous study [10] on paracetamol and morphine, it is estimated that 60% of all patients will have one or more NRS scores of more than 3. Using a simulation study, we calculated that to detect non-inferiority with a power of 75%, 200 patients (100 per group) would be required. Even though the power is 75%, this is considered to be sufficient for this secondary endpoint. This means that the sample size of 104 patients per group is sufficient for the non-inferiority analysis.

### Randomization, blinding, and treatment allocation

Blocked randomization with randomly chosen block size and stratification by center will be used. The randomization schedule will be kept in the local pharmacy at every center. To ensure concealed allocation, the pharmacist is the only person to have access to the randomization schedule. The randomization schedules are made by the study’s biostatistician. Study medication will be prepared at the participating centers. We will use standard morphine and paracetamol formulations.

In case of a medical emergency, the pharmacists can be consulted regarding what medication was administered to a patient.

## Statistical methods

The non-parametric Van Elteren test with stratification by center will be used to compare the primary outcome of cumulative age-adjusted morphine between groups.

Analysis of secondary outcomes will include length of PICU stay, number of hours on ventilation, and concomitant use of sedatives. These secondary outcomes will be compared by using linear regression analysis with group and treatment center as categorical independent variables. Analysis using linear regression models will be performed with cumulative rescue morphine (first 48 h) as outcome variable and group (morphine versus paracetamol) as predictor variable. Center, Down syndrome (yes/no), and cyanotic versus non-cyanotic cardiac defects will be added as covariates. Robust regression models will be used when necessary (i.e., when the outcome variable is non-normally distributed).

In a secondary, non-inferiority analysis, we will also compare the number of patients with one or more NRS scores of 4 or higher between the two groups by using a non-inferiority margin of 20%. Non-inferiority will be assessed by using a one-sided 97.5% confidence interval for the difference in the percentage of patients with at least one NRS score of 4 or higher between the paracetamol group and the morphine group, and non-inferiority will be proven if the upper limit of this confidence interval is lower than 20%. The confidence interval will be calculated by using the method of Klingenberg [60] with adjustment for center.

Adverse drug reactions will be specified as hemodynamic, gastrointestinal, respiratory reactions or pediatric delirium, as previously described. Adverse effects and other dichotomous outcomes, such as re-intubation, will be compared between groups by using Fisher’s exact tests, and the uncertainty in these estimated proportions will be assessed by using 95% confidence intervals. The level of significance will be set at 5%, and all tests will be two-sided. NONMEM will be used to perform population PK analysis.

### Safety

Patients can be withdrawn from the study at any time by the investigator or the treating physician. The intention-to-treat analysis will include all subjects. The subjects that have been withdrawn during the study will be included only for the time period in which they have participated. The cumulative morphine requirement will be calculated for the time that the patient participated in the study.

An external data safety monitoring board (DSMB) composed of pediatric intensivists and cardio-anesthesiologist with extensive clinical and research experience in the field of analgosedation in the PICU is established. The study protocol does not contain an interim analysis. The secondary endpoints require more included patients than the primary endpoint. An interim analysis could terminate the trial prematurely on the basis of a favorable primary outcome while not having enough power to assess secondary endpoints. However, the DSMB can advise us to stop the trial if necessary. The DSMB has advised the researchers during the study set-up and evaluated study proceedings after inclusion of the first 10 patients. The DSMB evaluated inclusion rate and safety of participants (need for rescue morphine in both groups) several times during the inclusion period, and the last meeting was in October 2017. The DSMB advised us to continue the study without changes to the protocol.

An advisory board composed of representatives from two patient and parent associations (*Stichting Kind en Ziekenhuis* and *Patientvereniging Aangeboren Hartafwijkingen*), a neonatologist and clinical pharmacologist, and a pediatric cardiologist is established. The advisory board has been involved in the design of the study and parental participation.

### Data management and monitoring

Data are collected through case report forms in OpenClinica, a web-based database (OpenClinica, LCC, Waltham, MA, USA), supported by the Erasmus MC. Range checks for data values were added in the Code of Federal Regulations (CRF), if possible, to promote data quality. Data management is coordinated by the Erasmus MC researchers and trial support unit. All participants receive a trial ID, and the personal details are known only to the researchers, pharmacy, and attending physician. Double data entry and validation are carried out by all participating centers. Monitoring of the data and trial proceedings is coordinated by the Erasmus MC. Monitoring of the data and trial proceedings is carried out per research site before the start of the trial, soon after the start of the trial, and three times a year for the duration of the trial. A close-out visit is carried out after completion of the inclusion period per research site. Trial auditing is carried out only by invitation from the hospital board and its frequency is not specified.

The Erasmus MC, as the coordinating center, owns intellectual property. The investigators have unlimited access to the final dataset. A newsletter with the study results will be made available on the website of the patient and parent organizations and will be sent to participants upon request. Public access to the data and data sharing is in line with the guidelines of ZonMw (data management plan), the main funder of this trial. Data access is restricted to authorized use only, and access will be granted by the researchers upon reasonable request.

## Discussion

PK-based dosing of analgesics in children after cardiac surgery is lacking. IV paracetamol as the primary analgesic after cardiac surgery could have an opioid-sparing effect and therefore fewer opioid-related adverse drug reactions. This study will also provide the necessary PK and PD parameters to establish a PKPD-based dosing regimen for analgesics after cardiac surgery. This will lead to a more individualized dosing regimen to guide clinicians in providing the best analgesic therapy for their patients.

The results of this trial will be incorporated in an international guideline for pain treatment after cardiac surgery in neonates and children who are 0–36 months old. This guideline will be endorsed by several scientific societies for pediatricians and anesthesiologists.

### Trial status

**Fig. 1:**
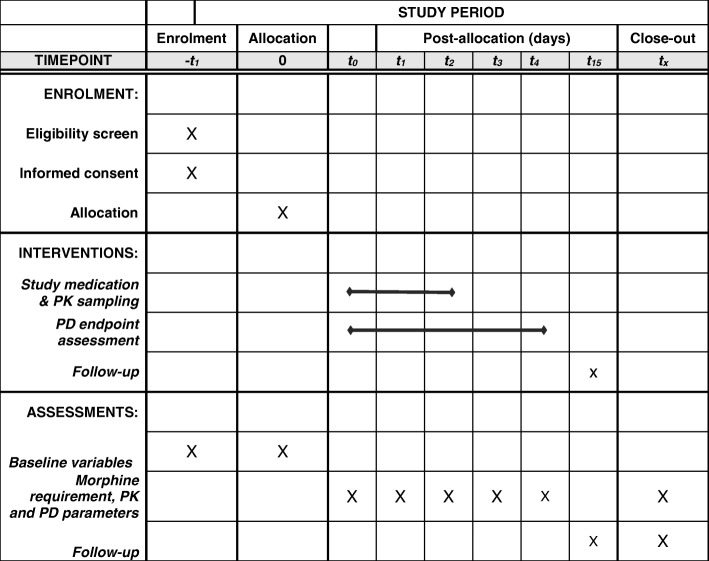
SPIRIT figure Pediatric Analgesia after Cardiac Surgery (PACS) study

The study was initiated in March 2016. On March 23, 2018, 124 patients had been enrolled in the study. Enrolment of all 208 patients is expected to be completed in the fall of 2018. The Standard Protocol Items Recommendations for Interventional Trials (SPIRIT) checklist and figure on the trial proceedings are added as Additional file 1 and Fig. 1, respectively.

### Additional files

Additional file 1: SPIRIT 2013 Checklist: Recommended items to address in a clinical trial protocol and related documents*. (DOC 121 kb)

Additional file 2: Short patient information flyer. (PDF 328 kb)

## Appendix

**Table 1:** Morphine dosing algorithm (adapted from Wang et al. [40])

Weight of patient, kg	Dosis, μg/kg per h	Dosis, μg/day	Continuous infusion, mL/h solution 100 μg/mL
2.5–2.9	3.9	257.4	0.1
3–3.4	5.3	413.4	0.2
3.5–3.9	7.1	639.0	0.3
4–4.4	9.3	948.6	0.4
4.5–4.9	11.4	1299.6	0.5
5–5.4	13.2	1663.2	0.7
5.5–5.9	14.5	2001.0	0.8
6–6.4	15.4	2310.0	1.0
6.5–6.9	16.0	2592.0	1.1
7–7.4	16.4	2853.6	1.2
7.5–7.9	16.6	3087.6	1.3
8–8.9	16.8	3427.2	1.4
9–9.9	16.9	3853.2	1.6
10–10.9	16.8	4233.6	1.8
11–11.9	16.7	4609.2	1.9
12–12.9	16.6	4980.0	2.1
13–13.9	16.5	5346.0	2.2
14–14.9	16.4	5707.2	2.4
15–15.9	16.0	5952.0	2.5
16–16.9	16.0	6336.0	2.6
17–17.9	16.0	6720.0	2.8
18–18.9	16.0	7104.0	3.0
19–19.9	16.0	7488.0	3.1
20–20.9	16.0	7872.0	3.3

## Abbreviations

CAPD: Cornell Assessment of Pediatric Delirium
COMFORT-B: COMFORT-Behavior Scale
CPB: Cardiopulmonary bypass
DSMB: Data Safety Monitoring Board
ICU: Intensive care unit
IV: Intravenous
NAPQI: N-acetyl-p-benzo-quinone imine
NISS: Nurse Interpretation of Sedation Score
NONMEM: Non-linear mixed-effect modeling
NRS: Numeric Rating Scale
PD: Pharmacodynamics
PICU: Pediatric intensive care unit
PK: Pharmacokinetics
SIRS: Systemic inflammatory response syndrome
SOS: Sophia Observation withdrawal Symptoms
SOS-PD: Sophia Observation withdrawal Symptoms–Pediatric Delirium
UMC: University Medical Center
UZ: University Hospital
