# Supplementary material for: Intravenous morphine versus intravenous paracetamol after cardiac surgery in neonates and infants: a study protocol for a randomized controlled trial
Source: Trials. 2018 Jun 13;19:318. doi: 10.1186/s13063-018-2705-5 (PMC5998570; doi:10.1186/s13063-018-2705-5)
Supplement: Supplementary file 2 — Short patient information flyer. (PDF 328 kb) [file 13063_2018_2705_MOESM2_ESM.pdf]

Heeft u nog vragen?

Op de website van de intensive care kinderen van het Erasmus MC-Sophia vindt u meer informatie over dit onderzoek. U vind hier ook informatie van patient verenigingen over kinderen met aangeboren hartafwijkingen en informatie over medisch wetenschappelijk onderzoek bij kinderen.

De dag voor de operatie komt de onderzoeker bij u langs op de medium care afdeling voor aanvullende informatie en om uw vragen te beantwoorden.

De onderzoekers van deze studie zijn:

Erasmus MC-Sophia kinderziekenhuis, Rotterdam:
Drs. G.A. Zeilmaker, arts, afdeling intensive care kinderen en thoraxchirurgie
Dr. E.D. Wildschut, kinderarts, afdeling intensive care kinderen

Wilhelmina kinderziekenhuis, Utrecht:
Drs. E. Koomen, anesthesist, afdeling intensive care kinderen
Dr. N.J.G. Jansen, kinderarts, afdeling intensive care kinderen

Beatrix kinderziekenhuis, Groningen:
Dr. M.C.J. Kneyber, kinderarts, afdeling intensive care kinderen

Universitair Ziekenhuis, Leuven, België:
Dr. S. Maebe, kinderarts, afdeling intensive care kinderen
Prof. dr. D.Vlasselaers, intensivist-anesthesist, afdeling intensive care kinderen

Voor vragen kunt u via email contact opnemen met:
E-mail: g.zeilmaker@erasmusmc.nl

Versie 2, augustus 2017. Protocol ID NL53085.078.15

Informatiefolder voor ouders

PACS study:
Pediatric Analgesia after Cardiac Surgery

Paracetamol of morfine als pijnstiller na een open hartoperatie

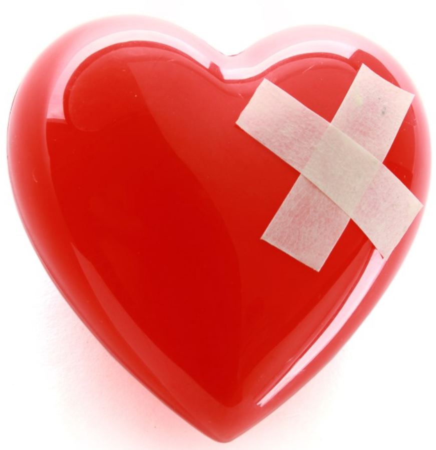

Augustus 2017

Paracetamol of morfine als pijnstiller na een open hartoperatie

PACS study

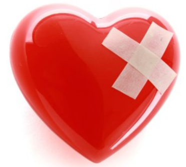

Geachte ouder/verzorger,

Uw kind heeft een hartaandoening en zal binnenkort hieraan moeten worden geopereerd.

Na de operatie wordt standaard morfine gegeven als pijnstiller. Dit middel werkt goed tegen de pijn, maar heeft ook bijwerkingen. Daarom zijn wij op zoek naar andere medicijnen die even goed werken tegen de pijn, maar zonder de bijwerkingen.

Uw kind komt in aanmerking voor deelname aan het onderzoek naar deze nieuwe pijnstiller.

Voordat u deze beslissing neemt is het uiteraard belangrijk dat u meer weet over het onderzoek.

In deze folder vindt u informatie over dit onderzoek.

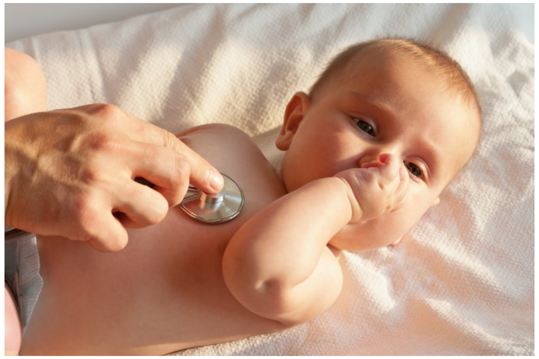

WAAROM DOEN WE DIT ONDERZOEK?

Morfine kan ongewenste effecten hebben op onder andere de bloeddruk of de ademhaling van uw kind. Uw kind kan ook last hebben van misselijkheid of obstipatie door de morfine. Paracetamol heeft deze bijwerkingen niet. Als paracetamol even goed werkt als morfine kunnen we kinderen in de toekomst vaker paracetamol als eerste keus pijnstiller geven na open hartoperaties.

WAT IS HET DOEL VAN HET ONDERZOEK?

We willen onderzoeken of paracetamol gegeven via het infuus even goed werkt als morfine na open hartoperaties. Uit eerder onderzoek is namelijk gebleken dat paracetamol via het infuus snel en goed werkt tegen pijn na operaties. We weten niet of dit ook zo is voor pijn na open hartoperaties.

DE UITVOERING VAN HET ONDERZOEK

Als u besluit om uw kind niet mee te laten doen hoeft u hier geen reden voor op te geven. Uw kind krijgt de behandeling die het anders ook zou krijgen.
Als u toestemming geeft wordt er voor de operatie geloot of uw kind paracetamol of morfine krijgt. Na de operatie zullen de verpleegkundigen regelmatig observeren of uw kind pijn heeft en hoeveel. U wordt zelf ook gevraagd om de mate van pijn bij uw kind te beoordelen. Tenslotte kent u uw kind het best. U, de artsen en de onderzoekers weten dan niet of uw kind morfine of paracetamol krijgt. Hierdoor zijn de observaties zo betrouwbaar mogenlijk. Als de paracetamol of morfine niet voldoende werkt krijgt uw kind morfine.

Uw kind krijgt de paracetamol of morfine tot 48 uur na de operatie. De pijnobservaties gaan daarna nog 48 uur door.

WAT IS ANDERS DAN DE NORMALE BEHANDELING?

Op sommige momenten tijdens en na de operatie wordt er een klein beetje bloed afgenomen voor het onderzoek. Het bloed wordt afgenomen uit een infuus en uw kind hoeft niet extra te worden geprikt. Uit de urine katheter wordt urine afgenomen om te testen hoeveel van de medicijnen wordt uitgeplast.

Nadat uw kind weer thuis is zal de onderzoeker na twee dagen opbellen en vragen hoe het met uw kind gaat. Ook wordt er dan een korte vragenlijst afgenomen over de pijnbeleving van uw kind na ontslag uit het ziekenhuis. Op deze manier onderzoeken we de directe effecten van de pijnstilling, als de latere effecten van de pijnstilling op het herstel van uw kind.
